# TRPV1 blockade restores the baroreflex control of renal sympathetic nerve activity in cisplatin‐induced renal injury in rats

**DOI:** 10.1113/EP092618

**Published:** 2025-05-11

**Authors:** Mohammed H. Abdulla, Ella Murphy, Lauren Mulcahy, Edward J. Johns

**Affiliations:** ^1^ Department of Physiology University College Cork Cork Ireland

**Keywords:** baroreflex, capsazepine, cisplatin, renal sympathetic nerve activity, TNF‐α

## Abstract

Renal injury is associated with inflammatory responses within the kidney which could involve activation of transient receptor potential vanilloid 1 (TRPV1) channels. This study investigated whether TRPV1 channels modulate baroreflex regulation of renal sympathetic nerve activity (RSNA) in a rat model of cisplatin‐mediated renal injury. Rats were anaesthetised and prepared for measurement of mean arterial pressure (MAP), heart rate (HR) and RSNA 4 days after a single i.p. dose of cisplatin (5 mg kg^−1^). RSNA and HR baroreflex gain curves (BRC) were generated and the decrease in RSNA to volume expansion was determined during intrarenal capsazepine (CPZ, 15 µg kg^−1^ h^−1^) infusion. In the cisplatin group (MAP: 85 ± 13 mmHg; HR: 328 ± 17 bpm; RSNA: 0.83 ± 0.41 µV s), the slope and maximum gain of the BRC were approximately 50% lower (*P* = 0.015–0.033) than the control group (MAP: 78 ± 12 mmHg; HR: 352 ± 27 bpm; RSNA: 0.57 ± 0.36 µV s). Intrarenal CPZ infusion in the cisplatin group restored the slope (0.15 ± 0.04 vs. 0.09 ± 0.02, *P* = 0.014) of the RSNA BRC to near normal values. The RSNA response to volume expansion in the cisplatin group was enhanced following CPZ compared to vehicle infusion (−24 ± 14% vs. 1.7 ± 39%, *P* = 0.015). Intrarenal tumour necrosis factor α (TNF‐α) infusion ( 2 µg kg^−1^ h^−1^) in normal rats decreased the slope of the BRC by 40% (*P* = 0.035) compared to vehicle infusion, which was slightly enhanced following intrarenal CPZ infusion. These findings demonstrate that TRPV1 channels contribute to the depressed baroreceptor control of RSNA in renal injury. Furthermore, the action of TNF‐α in disrupting the baroreflex control mechanism partially involves TRPV1 channels.

## Abstract (highlights)

- **What is the central question of this study?**
  Does TRPV1 blockade restore the baroreflex control of renal sympathetic nerve activity (RSNA) in an animal model of kidney injury?
- **What is the main finding and its importance?**
  CPZ, a competitive inhibitor of capsaicin activation of the TRPV1, restored the baroreflex control of RSNA following cisplatin‐induced renal injury. This study offers insight into mechanisms of impaired baroreflex control in renal injury.

## 1 INTRODUCTION

The transient receptor potential vanilloid 1 (TRPV1) is a non‐selective cation channel which non‐selectively binds to capsaicin and can be blocked by the competitive antagonist capsazepine (CPZ) (Jaffal et al., 2022; Mergler et al., 2011; Yang et al., 2019). TRPV1 channels are located throughout the nervous system and are important in the transduction of pain (Marrone et al., 2017). In the kidneys, TRPV1 channels are abundant in the afferent renal nerves of the renal pelvic wall mainly in the fibres located between the muscular layer and the uroepithelium (Wang et al., 2010). These channels act as mechano‐ or chemoreceptors when activated and can increase afferent renal nerve traffic which can reflexly change the level of sympathetic outflow, including that to the kidney (Feng et al., 2008; Zhu et al., 2007). A recent study utilising TRPV1 gene‐deleted rats showed an attenuated sympathoexcitation, hypertension and improved glomerular filtration in the 2‐kidney‐1‐clip (2K1C) rat model of renal injury and hypertension (Stocker & Sullivan, 2023). These observations suggested that TRPV1 channels can play an important role in blood pressure regulation by modulating renal sympathetic activity and hence neurally mediated tubular fluid reabsorption.

Evidence is available demonstrating increased sympathetic nerve activity during kidney disease in experimental animals and in humans (Converse et al., 1992; Grassi G et al., 2011; Ye et al., 2002). Meanwhile, there is undue activation of TRPV1 channels by locally generated neuropeptides as part of the inflammatory response in renal injury/disease which contributes to renal tubular damage (Lu et al., 2020). On the other hand, TRPV1 activation induces the release of inflammatory mediators, an effect which can be blocked by TRPV1 antagonists (Ma et al., 2012). The question arises as to whether the reflex regulation of renal sympathetic nerve activity (RSNA) is modulated by TRPV1 under conditions of kidney injury and inflammation.

Cisplatin, a widely used chemotherapeutic drug, is known to cause acute kidney injury (AKI) in both clinical and experimental settings (Alhoshani et al., 2017; Bellomo et al., 2012; McSweeney et al., 2021). Cisplatin‐induced renal injury is associated with altered baroreflex function in both humans (Viana‐Cardoso et al., 2011) and rodents (Khan et al., 2014; Silva et al., 2021; Tong et al., 2023). This altered baroreflex may contribute to the increased risk of cardiovascular accidents in patients undergoing treatment with cisplatin (Frye et al., 2018). The cisplatin renal injury model has also been reported to have upregulated kidney tissue tumour necrosis factor α (TNF‐α) mRNA as well as increased serum levels of TNF‐α (Deng et al., 2001; Ramesh & Reeves, 2002). Similarly, exogenously administered TNF‐α into the kidneys of normal rats resulted in an exaggerated afferent renal nerve response to hypertonic NaCl and a blunted baroreflex response to saline volume expansion (Abdulla et al., 2024, 2019). Hence, TNF‐α inhibition in rodent models of cisplatin‐induced renal injury was found to ameliorate kidney function parameters such as urea nitrogen and reduce histological evidence of injury (Ramesh & Reeves, 2002, 2004). Additionally, TNF‐α inhibition with the calcineurin inhibitor tacrolimus restored the arterial and cardiopulmonary baroreflexes in a rat model of cisplatin renal injury (Abdulla et al., 2019). However, it has not yet been established whether TNF‐α‐induced effects on the baroreflex in the cisplatin renal injury model are mediated by TRPV1 channels. An understanding of the role of TRPV1 channels in this specific context is critical for elucidating the interplay between renal injury, inflammation and autonomic regulation.

The purpose of the present study was to evaluate the contribution of TRPV1 channels in modulating the arterial and cardiopulmonary baroreflex regulation of RSNA normally and during cisplatin‐induced renal injury. A further aim was to examine whether the inflammatory mediator TNF‐α contributed to the derangement of the RSNA baroreflex responses through a mechanism that involved renal TRPV1 channels. This was done by infusing TNF‐α intrarenally and examining the baroreflex in the presence of TRPV1 blockade.

## 2 METHODS

### 2.1 Animals

Male Wistar rats (body weight: 250–360 g) were procured from a local supplier (Envigo, Bicester, UK) and housed at University College Cork's animal facility. The rats were provided with a standard diet (Harlan‐Teklad, Bicester, UK) and had access to water ad libitum. The animals were maintained under a 12‐h light/12‐h dark cycle at a room temperature of 20 ± 3°C with 40–70% humidity. All experimental procedures complied with the European Union Regulations (Protection of Animals Used for Scientific Purposes, No. 543 of 2012, as amended and Directive 2010/63/EU), and were approved by the Animal Experimentation Ethical Committee at University College Cork.

### 2.2 Renal injury induction

Renal injury was induced by administering a single dose of cisplatin at 5 mg kg^−1^ i.p. and the animals were taken for a non‐recovery study 4 days post‐administration. Previous studies have demonstrated that this dosage of cisplatin induces renal injury within 4 days, evidenced by significantly elevated serum urea and creatinine levels (Campbell & Al‐Nasser, 1996; Gordon & Gattone, 1986). Additionally, this cisplatin dosage has been shown to increase the expression of interleukin (IL)‐1α, mitogen‐activated protein kinase and TNF‐α genes. Histopathological examination revealed that cisplatin also caused acute tubular injury, renal interstitial congestion and inflammatory cell infiltration (Alhoshani et al., 2017).

### 2.3 TNF‐α treatment

A separate cohort of rats (*n* = 6) received an intrarenal infusion of TNF‐α at a dose of  2 µg kg^−1^ h^−1^ for 30 min. The high‐pressure baroreflex was assessed in the presence of intrarenal TNF‐α and the results were compared to those obtained in the presence of a background intrarenal infusion of CPZ (15 µg kg^−1^ h^−1^) (AlMarabeh et al., 2021).

### 2.4 Experimental groups

#### 2.4.1 Cisplatin study

##### Arterial pressure baroreflex

Group 1 (control, *n* = 9): this group received vehicle (saline, 2 mL, i.p.) 4 days prior to the non‐recovery experiment. The baroreflex gain curve for the relationship between RSNA or heart rate (HR) and mean arterial pressure (MAP) was generated during intrarenal infusion of CPZ (15 µg kg^−1^ h^−1^) and compared to the vehicle. This group serves as a control for comparison with Group 2 across both phases.

Group 2 (renal injury, *n* = 7): this group received cisplatin (5 mg kg^−1^, i.p.; Hospira, Lake Forest, IL, USA) 4 days prior to the non‐recovery experiment (Abdulla et al., 2019, 2016; Goulding & Johns, 2015). The baroreflex gain curve for the relationship between RSNA or HR and MAP was generated during intrarenal infusion of CPZ (15 µg kg^−1^ h^−1^) and compared to the vehicle.

##### Volume expansion experiment

Group 3 (control, *n* = 7): this group received vehicle (saline, 2 mL, i.p.) 4 days prior to the non‐recovery experiment. The renal sympathoinhibitory response to acute volume expansion was evaluated during intrarenal infusion of CPZ (15 µg kg^−1^ h^−1^) and compared to the vehicle. This group serves as a control for comparison with Group 4 across both phases.

Group 4 (renal injury, *n* = 7): this group received cisplatin (5 mg kg^−1^, i.p.) 4 days prior to the non‐recovery experiment. The renal sympathoinhibitory response to acute volume expansion was evaluated during intrarenal infusion of CPZ (15 µg kg^−1^ h^−1^) and compared to the vehicle.

#### 2.4.2 TNF‐α study

Group 5 (negative control, *n* = 5): this group underwent two consecutive phases of intrarenal infusion of vehicle (saline, 1 mL h^−1^). The baroreflex gain curve for the relationship between RSNA or HR and MAP was evaluated during both phases.

Group 6 (positive control, *n* = 5): this group underwent two consecutive phases of intrarenal infusion of TNF‐α ( 2 µg kg^−1^ h^−1^). The baroreflex gain curve for the relationship between RSNA or HR and MAP was evaluated during both phases.

Group 7 (TNF‐α + CPZ, *n* = 6): this group underwent two phases of intrarenal infusion. In the first phase, TNF‐α (2 µg kg^−1^ h^−1^) was administered alone. In the second phase, TNF‐α was co‐administered with CPZ (15 µg kg^−1^ h^−1^). The baroreflex gain curve for the relationship between RSNA or HR and MAP was evaluated during both phases.

**FIGURE 1:**
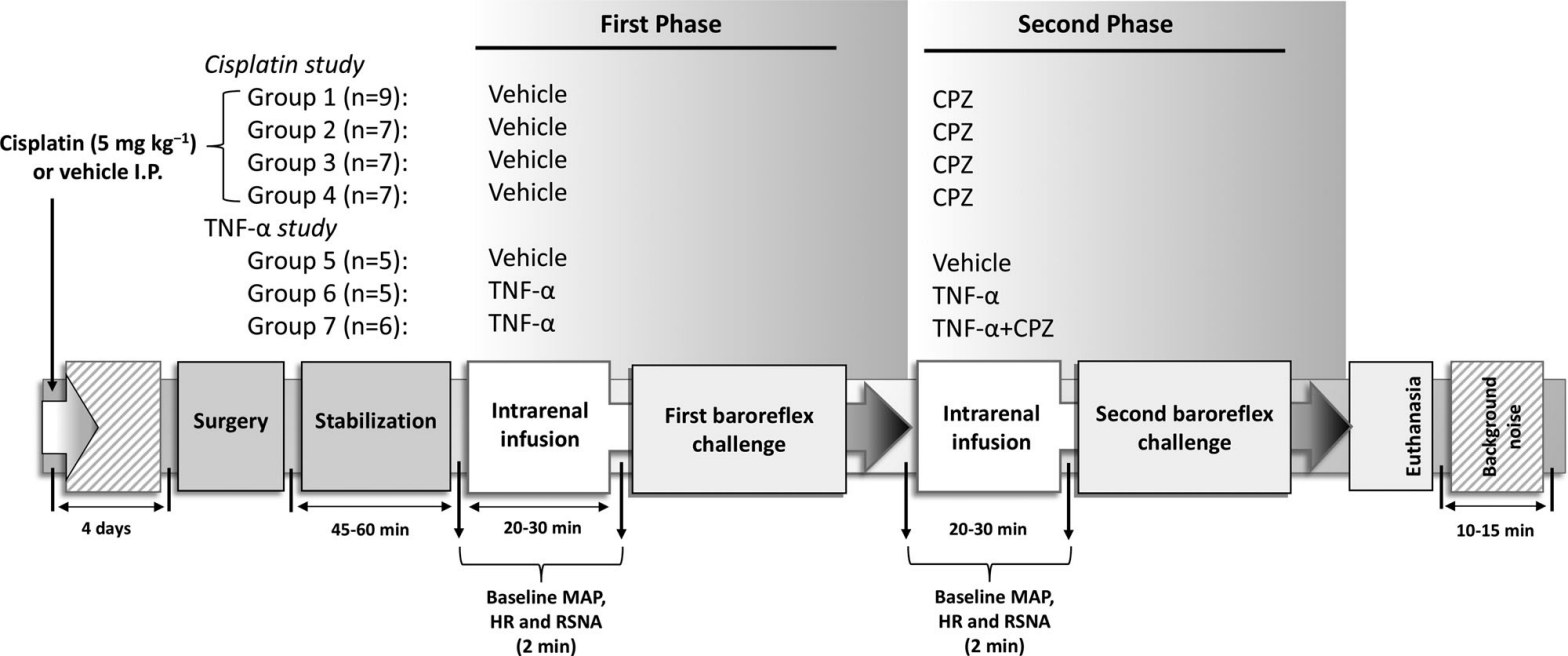
Summary of experimental timeline and interventions across all groups. AP, mean arterial pressure; CPZ, capsazepine; HR, heart rate; i.p., intraperitoneal; RSNA, renal sympathetic nerve activity.

Figure 1 provides an overview of the experimental timeline and interventions for all groups described above. The repeated vehicle and TNF‐α infusions were performed to account for potential time‐dependent changes in baroreflex sensitivity and to ensure that any observed effects were attributable to the intervention. The second phase of the TNF+CPZ group (Group 7) was compared with the second phase of the TNF group (Group 6) to evaluate the combined effect of TNF‐α and CPZ. Additionally, the second phase of the TNF group (Group 6) was compared with the second phase of the vehicle group (Group 5) to assess the effect of TNF‐α alone.

### 2.5 Surgical protocol

Rats were anaesthetised using a bolus injection of a mixture of α‐chloralose and urethane (16.5:250 mg ml^−1^ i.p.; Sigma‐Aldrich Co., Gillingham, UK) and supplemented with 0.3 mL kg^−1^ i.v. of the anaesthetic as required. A tracheotomy was carried out using a PE240 cannula (Portex, Hythe, UK) to maintain airway patency.

The right femoral artery was cannulated using a polyethylene PE25 cannula attached to a PE50 cannula. The infusion line contained heparinised saline (4 U ml^−1^) to maintain patency. This arterial cannula was connected to a fluid‐filled pressure transducer that was linked to a PowerLab data acquisition system (ADInstruments, Oxford, UK) for continuous recording of arterial blood pressure. Concurrently, the right femoral vein was cannulated using a PE50 cannula to facilitate the administration of maintenance doses of the anaesthetic and the continuous infusion of saline at a rate of 3 mL h^−1^ throughout the duration of the experiment.

A flank incision was used to expose the right kidney. A cannula (PP10, Portex) was inserted 4.5 mm to approximately the cortico‐medullary border of the right kidney and secured in place using Superglue (Abdulla et al., 2019, 2016). This allowed the infusion of saline (1 mL h^−1^) and CPZ (5 µg mL^−1^, Sigma‐Aldrich, St Louis, MO, USA) directly into the right kidney (AlMarabeh et al., 2021). The left kidney was exposed via a flank incision and the renal nerve bundle running between the renal artery and vein was dissected and placed on a bipolar multistranded stainless steel electrode (Medwire, Vernon, NY, USA). To prevent afferent nerve activity from the left kidney from interfering with the signal recording, the section of the renal nerve bundle distal to the electrodes and all other visible nerves in the area were crushed using surgical forceps. The nerve bundle and electrodes were then secured in place with a two‐compartment dental glue (Klasse 4 Dental, Augsburg, Germany), enabling continuous RSNA recording throughout the experiment. Data acquisition and processing were performed using LabChart 7 software (ADInstruments).

In the intrarenal TNF‐α study, the right kidney was exposed similarly to the cisplatin study described above. However, the corticomedullary cannula of the right kidney was used to infuse TNF‐α ( 2 µg kg^−1^ h^−1^, at 1 mL h^−1^, Sigma‐Aldrich, USA) as well as CPZ (15 µg kg^−1^ h^−1^  at 1 mL h^−1^) via an infusion pump (Hamilton, Kd Scientific, Holliston, MA, USA). The dose of TNF‐α was determined based on previous research conducted in our laboratory and other studies (Abdulla et al., 2019; Shahid et al., 2010; Sverrisson et al., 2015). The cannula was secured in place with Superglue, and the incision was closed using surgical stitches (4.0 Sutures, Ethicon, Livington, UK). A stabilisation period of at least 45 min was allowed post‐surgery before commencing the terminal study.

### 2.6 Experimental protocol

#### 2.6.1 Cisplatin study

##### Baseline values of MAP, HR and RSNA

The baseline values of MAP, HR and RSNA were recorded for 2 min following the stabilisation period and prior to any intervention.

##### Baroreflex gain curves of RSNA and HR

Following an intrarenal infusion of either vehicle (saline) or CPZ into the right kidney for a duration of 20 min, bolus doses of phenylephrine (PE; 50 µg mL^−1^, 0.2 mL) and sodium nitroprusside (SNP; 50 µg mL^−1^, 0.2 mL) were infused intravenously (i.v.) to increase and decrease blood pressure, respectively, and the first baroreflex gain curve was generated. To minimize the possible influence of each drug on the subsequent test, a recovery period of at least 5 min was allowed for MAP and HR to return to basal values before administering the next drug. Subsequently, a second baroreflex gain curve was generated in the presence of a continuous intrarenal infusion of CPZ.

##### The RSNA response to volume expansion in the cisplatin study

A saline infusion was commenced (0.25% body weight min^−1^ i.v.) and maintained for 30 min using an infusion pump (Graseby syringe pump 8100, Dublin, Ireland). A 5 min period of RSNA recordings was taken to provide baseline values which were averaged and taken as 100% values. The percentage reduction of RSNA from baseline values was then calculated for each 5 min period over the period of saline infusion. This was followed by a 30 min recovery period during which no saline was infused.

### 2.7 TNF‐α study

#### 2.7.1 Baroreflex gain curves of RSNA and HR

The experimental protocol for the TNF‐α study comprised two phases. The first phase involved an intra‐renal TNF‐α infusion and served as a control for the second phase which included intra‐renal infusion of either TNF‐α alone (group 5) or TNF‐α plus CPZ infusion (group 6). During the first phase, TNF‐α was infused into the right kidney at a rate of 1 mL h^−1^ for 30 min. During this period, PE and SNP were administered to generate baroreceptor gain curves. Subsequently, a mixture of TNF‐α and CPZ was infused into the right kidney for an additional 30 min, during which a second baroreceptor gain curve was generated. Baseline values for MAP, HR and RSNA were recorded for 2 min both prior to and following the intrarenal infusion of TNF‐α and CPZ. At the end of the experiment, the animals were euthanised with an overdose of anaesthetic (α‐chloralose–urethane mixture, 5 mL kg^−1^, i.v.) and the background noise for RSNA was recorded for 10–15 min and the average of the last 2 min was calculated and subtracted from all RSNA measurements.

### 2.8 Data analysis

RSNA was recorded with a high‐impedance head stage attached to an amplifier (NeuroAMP EX, ADInstruments) connected to a PowerLab data‐acquisition system. The RSNA signal was recorded according to the following parameters: an amplification of 10,000×, high‐pass filter of 100 Hz, low‐pass filter of 2 kHz and a sampling rate of 1 kHz. The data were processed using LabChart 7 software (ADInstruments) and stored on a computer hard disk for later analysis.

The baroreflex gain curve was constructed off‐line using changes in MAP, HR and RSNA in response to PE and SNP. LabChart data points before (120 s), during (220 s) and after (120 s) each stimulus were selected and transferred to a Microsoft Excel spreadsheet in which data points were reduced by 1000× so that there were 920 points for MAP, HR and RSNA for each rat. The data points were further reduced by binning the RSNA and HR data for every 5‐mmHg change in MAP using a pivot table. The resulting data points were then transferred to GraphPad Prism 6 software (GraphPad Software Inc., La Jolla, CA, USA) and the baroreflex gain curve was generated using these points by utilising a four‐parameter logistic equation (*y *= *A*_1_/[1 + exp(*A*_2_ [*x* − *A*_3_])] + *A*_4_), where *y* is RSNA or HR, *A*_1_ is the response range over which baroreceptors operate, *A*_2_ is the slope or gain coefficient, *A*_3_ is midpoint blood pressure and *A*_4_ is the minimum response of RSNA or HR. The averaged *A*_1_–*A*_4_ data for the group were used to plot a mean curve. Additional baroreflex gain curve variables were calculated according to the following equations as reported by Miki et al. (2003):

$$\mathrm{Maximal}\mathrm{gain}=-A_{1}\times A_{2/4},$$

$$\mathrm{Maximum}\mathrm{response}=A_{1}+A_{4},$$

$$\mathrm{MAP}\mathrm{threshold}=-2.0/A_{2}+A_{3},$$

$$\mathrm{MAP}\mathrm{saturation}=2.0/A_{2}+A_{3},$$

Operatingrange=MAPsaturation−MAPthreshold.

### 2.9 Statistical analysis

The baseline values of MAP, HR and RSNA, in addition to high‐pressure baroreflex gain curve parameters for all groups, were analysed using repeated‐measures two‐way analysis of variance (ANOVA) followed by a Bonferroni's or Tukey's *post hoc* test where relevant. During the volume expansion and recovery periods, 5‐min bins of data were averaged for all rats in the group and plotted against time. Comparisons of the reduction in RSNA due to volume expansion 25–30 min after starting i.v. saline infusion were analysed using repeated‐measures two‐way ANOVA followed by Bonferroni's *post hoc* test using GraphPad Prism 6 software for comparison between groups. Data are presented as means ± SD and a value of *P* < 0.05 was considered statistically significant.

## 3 RESULTS

### 3.1 Baseline MAP, HR and RSNA during intrarenal TRPV1 blockade

**FIGURE 2:**
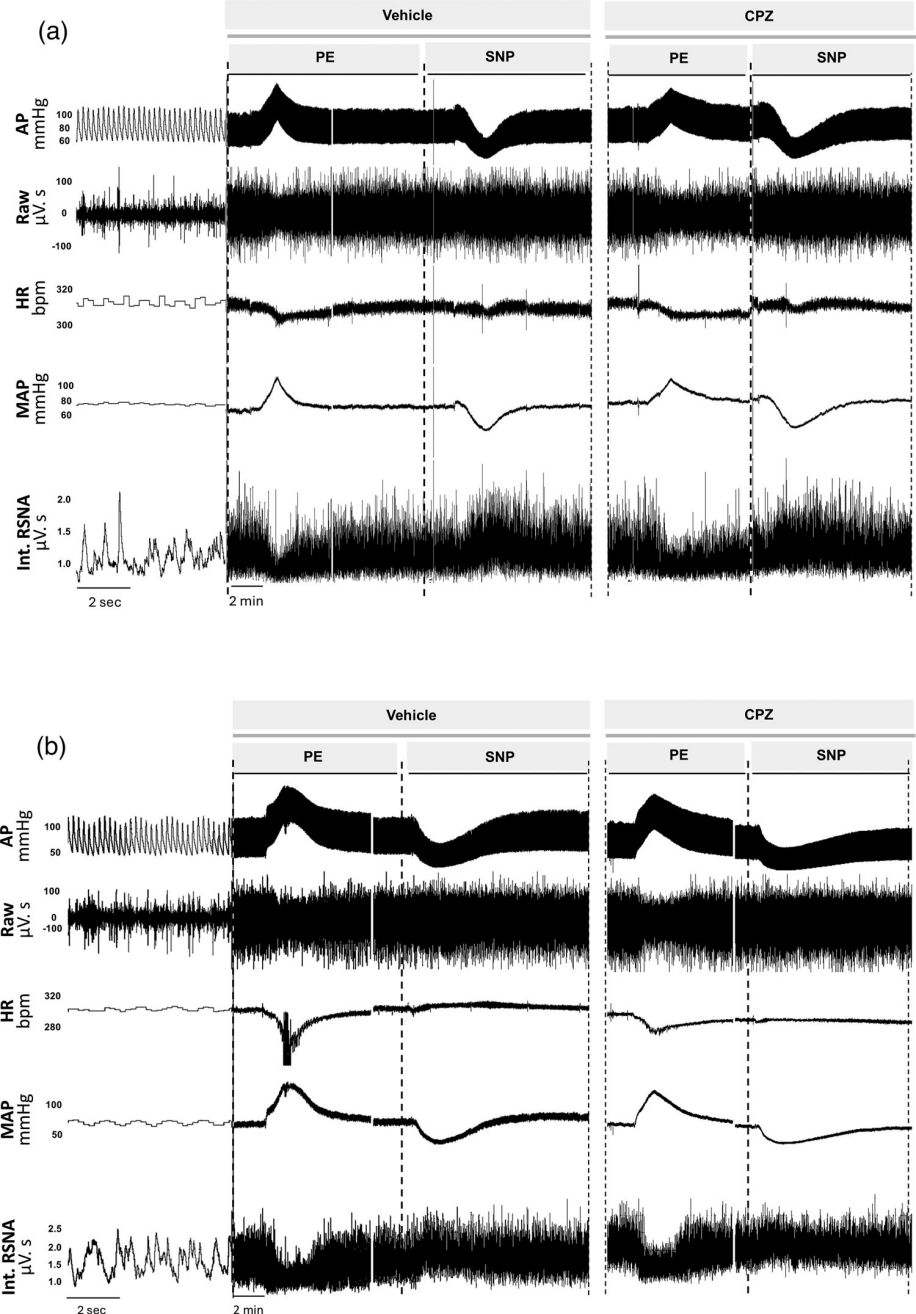
Sample data of baroreflex gain experiment in an individual control (a) and cisplatin (b) treated rat during intrarenal vehicle and CPZ infusion. Representative examples showing original recordings of pulsatile AP, the raw signal of RSNA, HR, MAP and integrated RSNA (Int. RSNA) responses to intravenous injections of PE and SNP. AP, arterial pressure; bpm, beats per minute; CPZ, capsazepine; HR, heart rate; MAP, mean arterial pressure; PE, phenylephrine; RSNA, renal sympathetic nerve activity; SNP, sodium nitroprusside.

**FIGURE 3:**
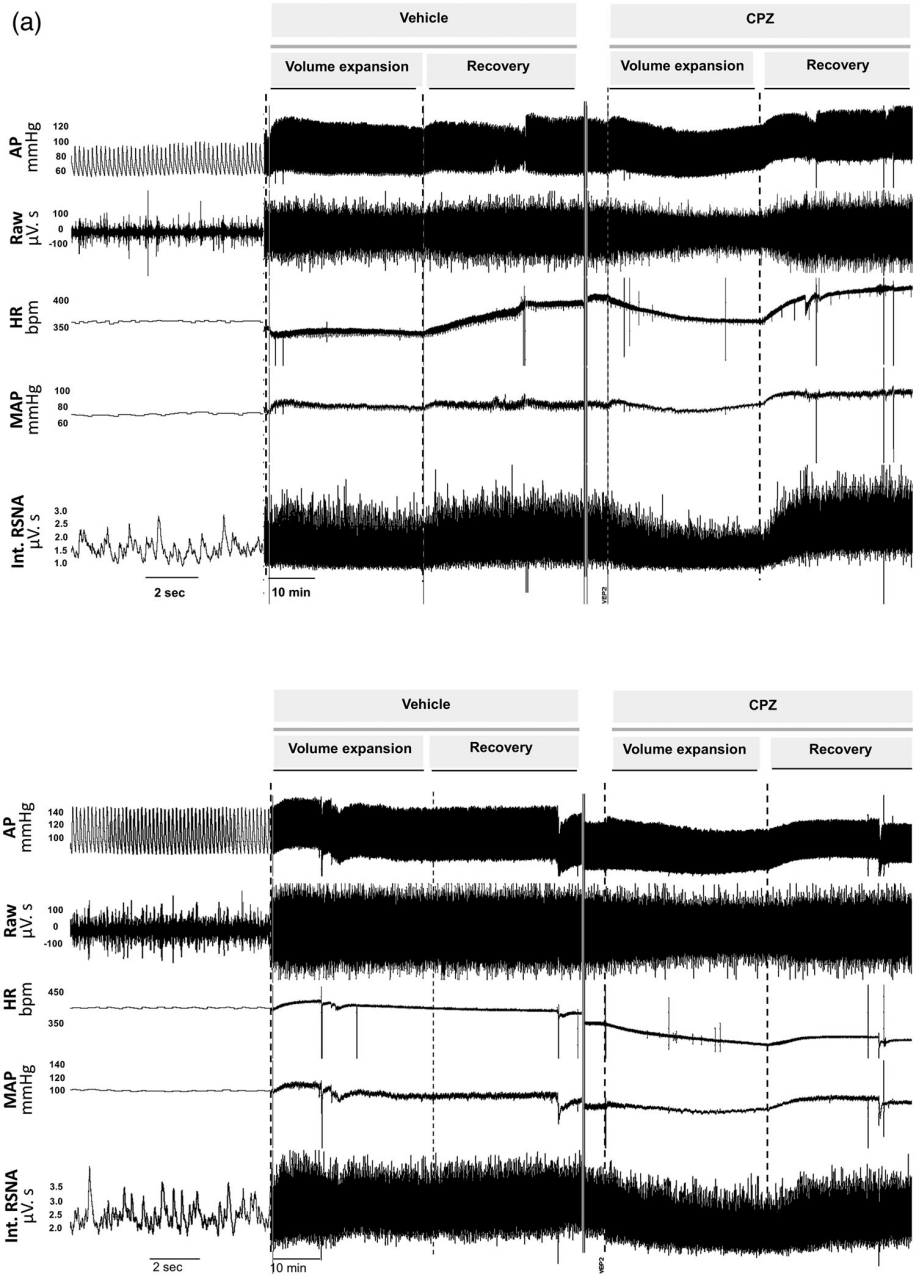
Sample data of volume expansion experiment in an individual control (a) and cisplatin (b) treated rat during intrarenal vehicle and CPZ infusion. Representative example showing original recordings of pulsatile AP, the raw signal of RSNA, HR, MAP and integrated RSNA (Int. RSNA) responses to intravenous injections of saline. AP, arterial pressure; bpm, beats per minute; CPZ, capsazepine; HR, heart rate; MAP, mean arterial pressure; RSNA, renal sympathetic nerve activity.

A representative recording of baseline parameters is shown in Figures 2 and 3. Baseline MAP, HR and RSNA parameters in cisplatin‐treated rats (Group 2) were not different from their respective baseline values in control rats (Group 1). Similarly, intrarenal TRPV1 blockade using CPZ had no effect on MAP, HR or RSNA values in either the cisplatin or control groups (Table 1).

**TABLE 1:** Baseline values of MAP, HR and RSNA in control and cisplatin groups.

	Intrarenal infusion					
	Vehicle		CPZ		Vehicle vs. CPZ	
Group	Before	During	Before	During	Before	During
Control (*n* = 9)						
MAP (mmHg)	78 ± 12	80 ± 12	84 ± 12	84 ± 13	*P* = 0.131	*P* = 0.260
HR (bpm)	352 ± 27	352 ± 35	355 ± 40	358 ± 41	*P* = 0.948	*P* = 0.691
RSNA (µV s)	0.57 ± 0.36	0.63 ± 0.38	0.85 ± 0.50	0.90 ± 0.50	*P* = 0.198	*P* = 0.161
Cisplatin (*n* = 7)						
MAP (mmHg)	85 ± 13	87 ± 14	94 ± 11	93 ± 11	*P* = 0.103	*P* = 0.308
HR (bpm)	328 ± 17	327 ± 18	328 ± 20	334 ± 26	*P* = 0.940	*P* = 0.431
RSNA (µV s)	0.83 ± 0.41	0.87 ± 0.42	0.90 ± 0.54	1.0 ± 0.64	*P* = 0.696	*P* = 0.667
	**Control vs. cisplatin**		**Control vs. cisplatin**			
	*P* = 0.644	*P* = 0.576	*P* = 0.202	*P* = 0.291		
	*P* = 0.303	*P* = 0.199	*P* = 0.803	*P* > 0.999		
	*P* = 0.412	*P* = 0.461	*P* > 0.999	*P* > 0.999		

*Note*: Values were recorded for 2 min directly before and then 20 min after intrarenal saline infusion prior to the first baroreflex gain curve. Similarly, values were recorded for 2 min immediately before and then 20 min after intrarenal CPZ infusion prior to the second baroreflex gain curve. Comparisons between control and cisplatin groups, and between vehicle and CPZ were carried out using repeated‐measures two‐way ANOVA followed by Bonferroni's and Tukey's *post hoc* tests where relevant. Abbreviations: ANOVA, analysis of variance; bpm, beats per minute; CPZ, capsazepine; HR, heart rate; MAP, mean arterial pressure; RSNA, renal sympathetic nerve activity.

Furthermore, there were no changes in MAP, HR or RSNA before or during the first and second intrarenal infusion of the vehicle (Group 5). In contrast, during TNF‐α infusion (Group 6), there was a significant (*P *< 0.001) increase in RSNA during the second intrarenal infusion of TNF‐α. However, the intrarenal infusion of CPZ in the presence of a background TNF‐α infusion (Group 7) showed no changes in RSNA, MAP or HR (Table 2 and Table 3) compared to the infusion of TNF‐α alone.

**TABLE 2:** Baseline values of MAP, HR and RSNA in vehicle, TNF‐α and TNF‐α + CPZ groups.

	Intrarenal infusion					
Group	Before	During	Before	During	Before vs. During	
	**Vehicle**		**Vehicle**			
Vehicle (*n* = 5)						
MAP (mmHg)	86 ± 5	87 ± 7	87 ± 7	87 ± 7	*P* = 0.974	p > 0.999
HR (bpm)	349 ± 30	351 ± 37	350 ± 40	349 ± 40	*P* > 0.999	*P* = 0.872
RSNA (µV s)	0.70 ± 0.33	0.57 ± 0.29	0.59 ± 0.37	0.58 ± 0.36	*P* = 0.833	*P* = 0.998
	**TNF‐α**		**TNF‐α**			
TNF‐α (*n* = 5)						
MAP (mmHg)	82 ± 11	82 ± 13	97 ± 11	97 ± 11	*P* = 0.190	*P* = 0.264
HR (bpm)	364 ± 60	362 ± 60	391 ± 54	393 ± 53	*P* = 0.105	*P* = 0.060
RSNA (µV s)	0.66 ± 0.28	0.68 ± 0.29	0.86 ± 0.28	0.82 ± 0.28	***P* < 0.001**	***P* < 0.0001**
	**TNF‐α**		**TNF‐α+CPZ**			
TNF‐α + PZ (*n* = 6)						
MAP (mmHg)	82 ± 9	85 ± 16	84 ± 16	88 ± 14	*P* = 0.855	*P* = 0.738
HR (bpm)	366 ± 25	361 ± 19	364 ± 30	358 ± 27	*P* = 0.996	*P* = 0.961
RSNA (µV s)	0.63 ± 0.18	0.57 ± 0.09	0.63 ± 0.08	0.68 ± 0.11	*P* > 0.999	*P* = 0.122
	**Vehicle vs. TNF‐α** ^a^		**TNF‐α vs. TNF‐ α+CPZ** ^a^			
	*P* = 0.367	*P* = 0.383	*P* = 0.174	*P* = 0.399		
	*P* = 0.265	*P* = 0.216	*P* = 0.526	*P* = 0.354		
	*P* = 0.249	*P* = 0.328	*P* = 0.334	*P* = 0.659		

*Note*: Values were recorded for 2 min directly before and then 20 min after intrarenal TNF‐α infusion prior to the first baroreflex gain curve. Similarly, values were recorded for 2 min immediately before and then 20 min after intrarenal vehicle, TNF‐α or TNF‐α + CPZ infusion prior to the second baroreflex gain curve. For each paramter, comparisons were made between values recorded during vs before intrarenal administration of drugs or vehicle. ^a^denotes comparisons made between groups at the same time point (i.e., before vs. before and during vs. during) during the second intrarenal infusion phase. Comparisons between vehicle, TNF‐α and TNF‐α + CPZ groups were carried out using repeated‐measure two‐way ANOVA followed by Bonferroni's and Tukey's *post hoc* tests where relevant. Significance was taken at *P* < 0.05. Abbreviations: ANOVA, analysis of variance; bpm, beats per minute; CPZ, capsazepine; HR, heart rate; MAP, mean arterial pressure; RSNA, renal sympathetic nerve activity.

**TABLE 3:** Logistic model and derived variables describing the baroreflex curves for RSNA in control and cisplatin groups.

	Intrarenal infusion		Vehicle vs. CPZ	
Group	Vehicle	CPZ		
Control (*n* = 9)				
*A*_1_ (%)	89 ± 19	93 ± 13	*P* = 0.935	
*A*_2_ (%RSNA mmHg^−1^)	0.14 ± 0.04	0.11 ± 0.03	*P* = 0.362	
*A*_3_ (mmHg)	93 ± 13	92 ± 11	*P >* 0.999	
*A*_4_ (%)	39 ± 11	38 ± 10	*P >* 0.999	
Max gain (%RSNA mmHg^−1^)	−2.99 ± 0.90	−2.65 ± 0.76	*P* = 0.741	
Maximum response (%)	128 ± 13	131 ± 8	*P >* 0.999	
MAP threshold (mmHg)	78 ± 14	73 ± 13	*P* = 0.613	
MAP saturation (mmHg)	109 ± 12	111 ± 11	*P* = 0.569	
Operating range (mmHg)	31 ± 7	38 ± 12	*P* = 0.242	
Cisplatin (*n* = 7)				
*A*_1_ (%)	74 ± 25	68 ± 31	*P* = 0.692	
*A*_2_ (%RSNA mmHg^−1^)	0.09 ± 0.02	0.15 ± 0.04	*P* = **0.014**	
*A*_3_ (mmHg)	105 ± 12	98 ± 10	*P* = 0.059	
*A*_4_ (%)	51 ± 28	50 ± 20	*P > *0.999	
Max gain (%RSNA mmHg^−1^)	−1.70 ± 0.91	−2.53 ± 1.4	*P* = 0.125	
Maximum response (%)	125 ± 11	117 ± 12	*P* = 0.378	
MAP threshold (mmHg)	81 ± 13	84 ± 9	*P >* 0.999	
MAP saturation (mmHg)	128 ± 13	112 ± 11	***P <* 0.0001**	
Operating range (mmHg)	47 ± 8	29 ± 7	*P* = **0.003**	
	**Control vs. cisplatin**	**Control vs. cisplatin**		
	*P* *= 0.381*	*P* = 0.055		
	*P* = **0.015**	*P* = 0.111		
	*P* = 0.099	*P* = 0.674		
	*P *> 0.999	*P *> 0.999		
	*P* = **0.033**	*P *> 0.999		
	*P *> 0.999	*P* = **0.032**		
	*P *> 0.999	*P* = 0.231		
	*P* = **0.005**	*P *> 0.999		
	*P* = **0.003**	*P* = 0.096		

*Note*: Values are means ± SD. *A*_1_, response range; *A*_2_, gain coefficient; *A*_3_, pressure at the midrange of the curve (centring point); *A*_4_, minimum response. Comparisons between control and cisplatin groups, and between vehicle and CPZ were carried out using repeated‐measure two‐way ANOVA followed by Bonferroni's *post hoc* test. Significance was taken at *P* < 0.05. Abbreviations: CPZ, capsazepine; HR, heart rate; MAP, mean arterial pressure; RSNA, renal sympathetic nerve activity.

For the volume expansion studies, there were no significant differences in baseline values for MAP, HR and RSNA following either intrarenal vehicle (saline) or CPZ infusion in control (group 3) or cisplatin‐treated (group 4) groups (Table S1: https://figshare.com/s/51ea85fc5ca7220ce1a1).

### 3.2 Arterial pressure baroreflex during intrarenal TRPV1 blockade

Representative recordings from arterial pressure baroreflex trials are shown in Figure 2. The average slope (gain coefficient) of the RSNA baroreflex function curve in cisplatin‐treated rats was lower (*P* = 0.015) than that of control rats. This was related to a significantly lower maximum gain (*P* = 0.033) but a higher operating range (*P* = 0.003) and MAP saturation (*P* = 0.005) in the cisplatin‐treated rats compared to control (Table 3: Significance was taken at *P* < 0.05).

**FIGURE 4:**
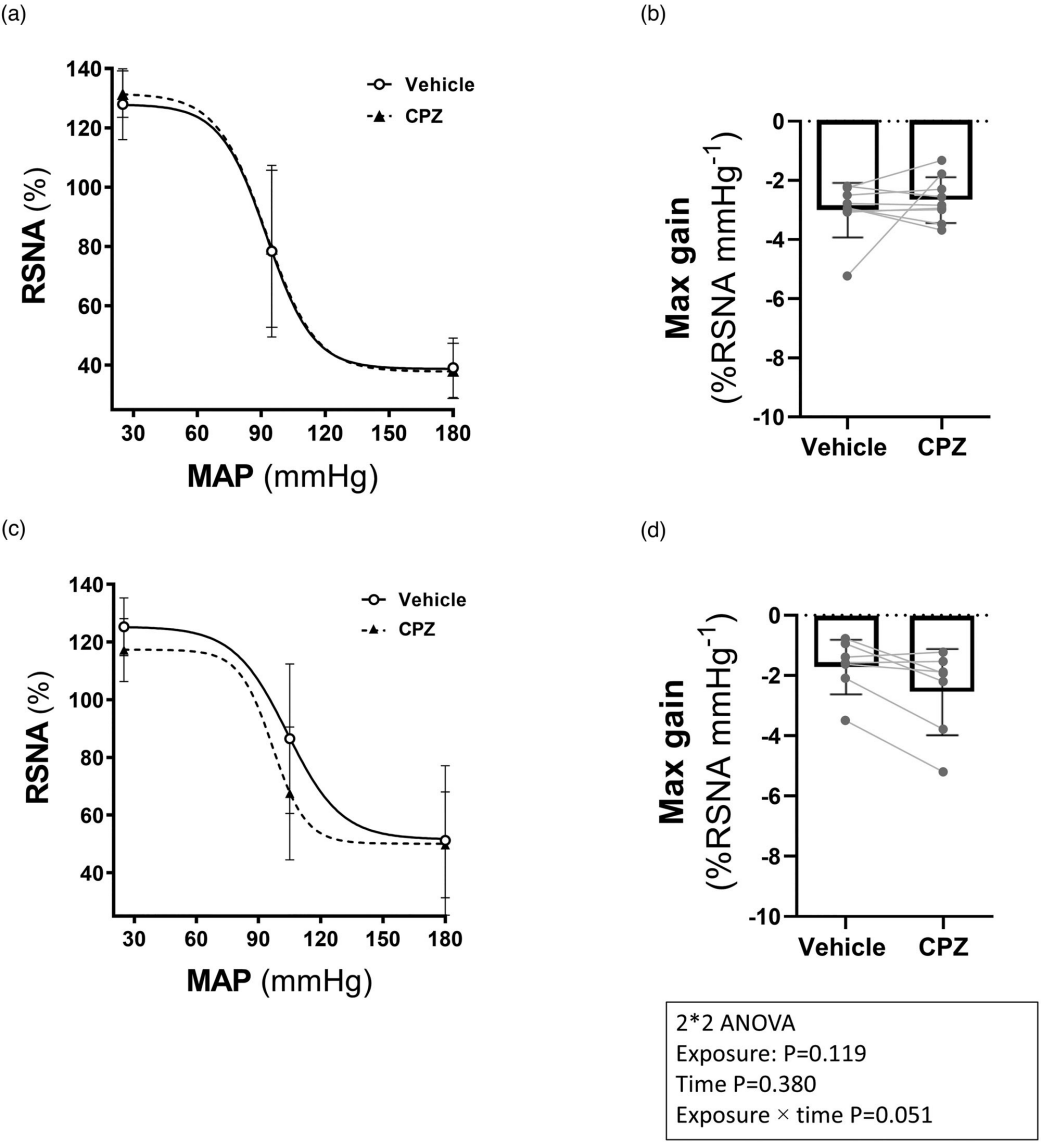
Average BRC and maximal gain of RSNA during intrarenal vehicle (saline infusion followed by CPZ in control (*n* = 9) (a, b) and cisplatin (*n* = 7) (c, d) groups. The baseline value of RSNA was taken as 100%. Symbols on the baroreflex curves (a, c) represent the highest, mid and lowest point values for vehicle (continuous line and open circles) and CPZ (dashed line and filled triangles). Symbols on the maximum gain bar chart (b, d) represent individual rat points. BRC, baroreflex gain curves; CPZ, capsazepine.

During intrarenal infusion of CPZ, there were no changes in the arterial pressure baroreflex parameters compared to vehicles in the control group (Figure 4). However, the slope (gain coefficient) of the baroreflex gain curve in the presence of intrarenal CPZ in the cisplatin‐treated rats was significantly higher (*P* = 0.014) than vehicle. Additionally, both MAP saturation (*P *< 0.0001) and operating range (*P* = 0.003) in the cisplatin‐treated rats were decreased in the presence of intrarenal CPZ infusion compared to vehicle (Table 3).

**FIGURE 5:**
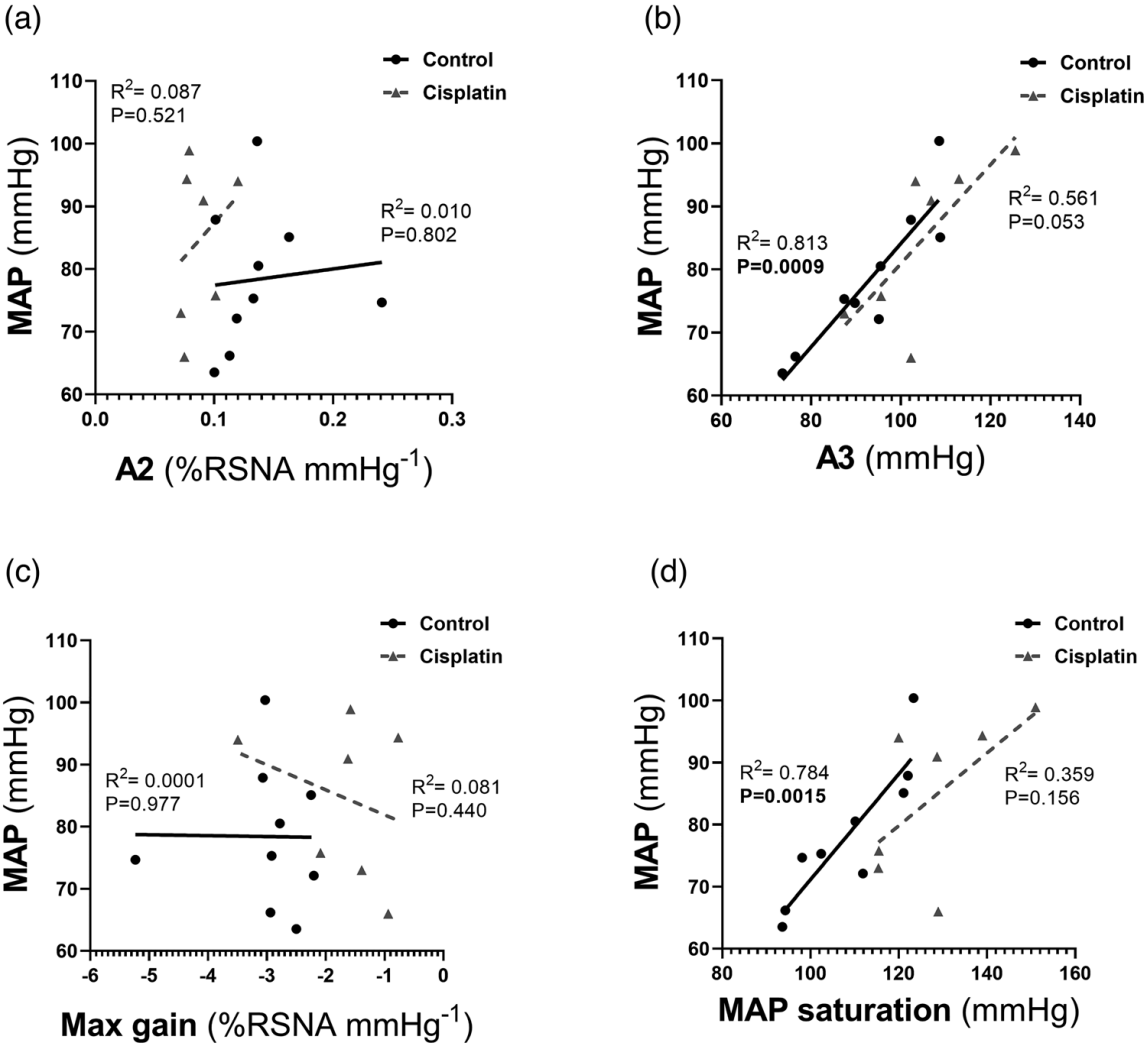
Correlations between baseline MAP and baroreflex parameters. The relationships between MAP and baroreflex parameters gain coefficient (*A*_2_; a), midpoint pressure of the response (*A*_3_; b), maximum gain (c) and MAP saturation (d) are shown for control (*n* = 9) and cisplatin (*n* = 7) rats. Linear regression analysis was performed. Significance was taken at *P* < 0.05. MAP, mean arterial pressure.

There was a significant correlation between baseline blood pressure and the midpoint pressure (*A*_3_ parameter, *R* ^2^ = 0.813, *P* = 0.0009) and MAP saturation (*R* ^2^ = 0.784, *P* = 0.0015) for the RSNA baroreflex function curves in the control group, but not in the cisplatin group. However, in either the control or cisplatin group, there was no significant correlation between baseline blood pressure and the slope coefficient (*A*_2_) or the maximum gain (Figure 5).

Intrarenal CPZ infusion did not affect the HR baroreflex curve parameters in cisplatin‐treated or control rats (Supporting information, Table S2: https://figshare.com/s/51ea85fc5ca7220ce1a1; Figure S1: https://figshare.com/s/1f3cfbd9060f3b0d61ac). However, the maximum response of the HR baroreflex curve was decreased (*P* = 0.037) in the presence of intrarenal CPZ infusion.

### 3.3 Volume expansion during intrarenal TRPV1 blockade

**FIGURE 6:**
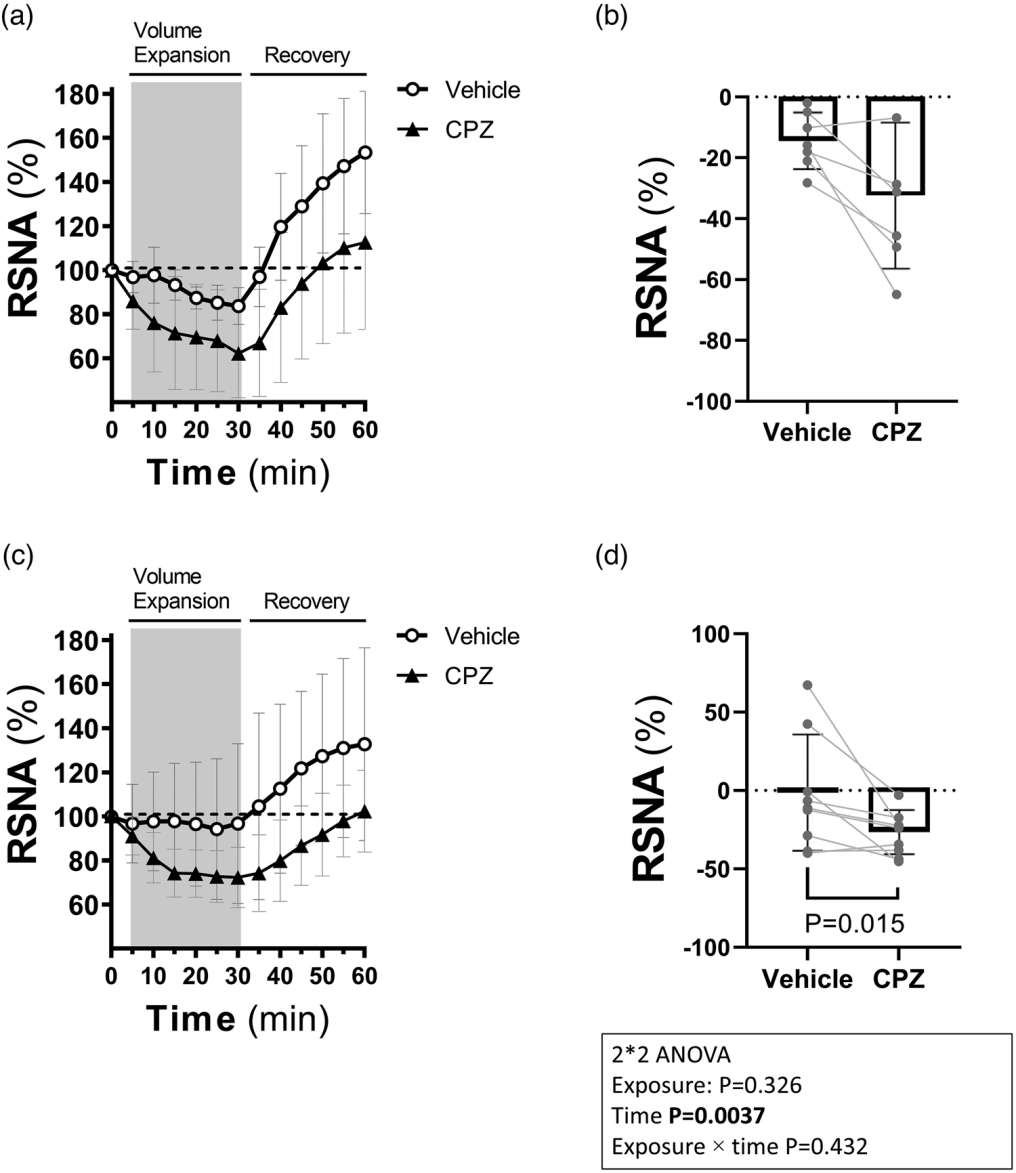
Time course (a) and percentage decrease (b) of the RSNA in response to volume expansion during intrarenal vehicle (saline) or CPZ infusion in control (*n* = 9) and cisplatin groups (*n* = 7). For the time course curve (a, c), the baseline value of RSNA was taken as 100% and the reduction in RSNA due to volume expansion was the average value for every 5‐min interval during volume load infusion and the recovery period. The percentage decrease in RSNA at the last point of volume expansion (25–30 min) was used to compare the magnitude of sympathoinhibition between the control and cisplatin groups (b, d). Analysis between vehicle and CPZ and between control and cisplatin groups was performed using repeated‐measures two‐way ANOVA followed by Bonferroni's *post hoc* test. Symbols on the RSNA bar chart (b, d) represent individual rat points. Significance was taken at *P* < 0.05. CPZ, capsazepine; RSNA, renal sympathetic nerve activity

Figure 6 illustrates the time course of the reflex renal sympathoinhibition due to volume expansion in the control and cisplatin groups. Although the decrease in RSNA in response to volume expansion in the cisplatin group was blunted compared to the control group (1.72 ± 39% vs. −14.33 ± 9%), it did not reach statistical significance (Figure 6). However, a potentiation of the RSNA sympathoinhibitory response to volume expansion was observed in cisplatin‐treated rats during intrarenal CPZ infusion (−24.35 ± 14% vs. 1.72 ± 39%, *P* = 0.015).

### 3.4 Arterial pressure baroreflex during TRPV1 blockade in the presence of intrarenal TNF‐α

**FIGURE 7:**
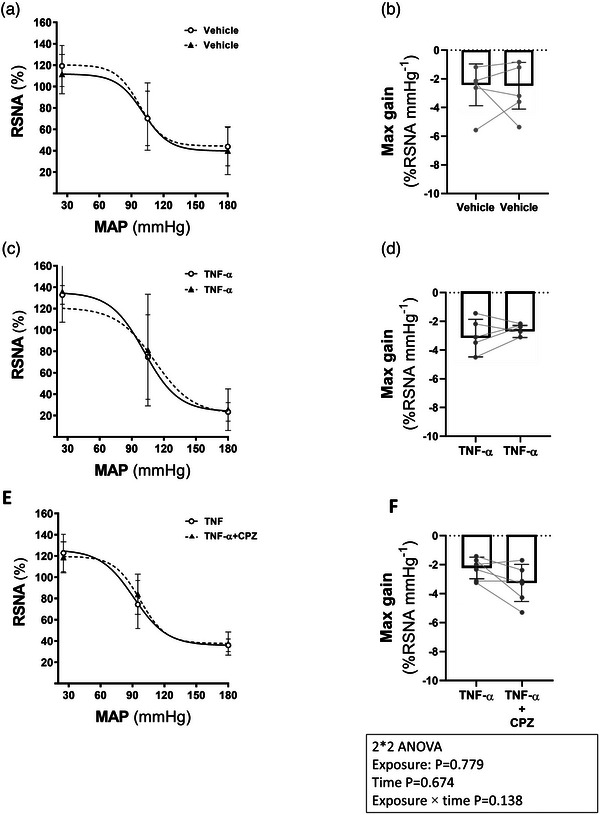
Average BRC and maximal gain of RSNA during intrarenal vehicle (*n* = 5; a, b), TNF‐α (*n* = 5; c, d) and TNF‐α+CPZ (*n* = 6; e, f) infusion. The baseline value of RSNA was taken as 100%. Symbols on the baroreflex curves (a, c, d) represent highest, mid and lowest point values for Vehicle and TNF‐α infusion (continuous line and open circles) and Vehicle, TNF‐α and TNF‐α + CPZ (dashed line and filled triangles). Symbols on the maximum gain bar chart (b, d, f) represent individual rat points. BRC, baroreflex gain curves; CPZ, capsazepine; RSNA, renal sympathetic nerve activity.

The effect of intrarenal infusion of CPZ on the baroreflex gain curves (BRC) generated in the presence of intrarenal TNF‐α is shown in Table 4 and Figure 7. Figure 7 demonstrates the averaged BRC and the maximal gain of the relationship between RSNA and MAP in the vehicle (Group 5), TNF‐α (Group 6) and TNF‐α + CPZ (Group 7) groups. In the group of rats where vehicle or TNF‐α was infused during the first and second intrarenal infusions (Table 4), there were no significant changes in the baroreflex gain curve parameters during the second compared to the first intrarenal infusion. However, the mid‐point MAP values during the second TNF‐α infusion were higher (*P* = 0.010) than during the first TNF‐α infusion. CPZ infusion during the second intrarenal TNF‐α infusion increased the slope (gain coefficient) of the baroreflex gain curve by 60% compared to TNF‐α alone. However, this increase in the gain coefficient was not statistically significant (Table 4).

**TABLE 4:** Logistic model and derived variables describing the baroreflex curves for RSNA in vehicle, TNF‐α and TNF‐α + CPZ groups.

Group	Intrarenal infusion		
Vehicle (*n* = 5)	Vehicle	Vehicle	Vehicle vs. Vehicle
*A*_1_ (%)	73 ± 29	70 ± 29	*P* > 0.999
*A*_2_ (%RSNA mmHg^−1^)	0.19 ± 0.05	0.17 ± 0.06	*P* > 0.999
*A*_3_ (mmHg)	98 ± 9	102 ± 13	*P* = 0.558
*A*_4_ (%)	45 ± 18	42 ± 23	*P* > 0.999
Max gain (%RSNA mmHg^−1^)	−2.76 ± 1.66	−2.84 ± 1.86	*P* > 0.999
Maximum response (%)	119 ± 19	112 ± 18	*P* > 0.999
MAP threshold (mmHg)	85 ± 8	87 ± 15	*P* > 0.999
MAP saturation (mmHg)	117 ± 19	122 ± 22	*P* > 0.999
Operating range (mmHg)	33 ± 16	35 ± 23	*P* > 0.999
**TNF‐α (*n* = 5)**	**TNF‐α**	**TNF‐α**	**TNF‐α vs. TNF‐α**
*A*_1_ (%)	112 ± 10	106 ± 20	*P* > 0.999
*A*_2_ (%RSNA mmHg^−1^)	0.11 ± 0.05	0.10 ± 0.02	*P* > 0.999
*A*_3_ (mmHg)	105 ± 20	115 ± 15	***P* < 0.010**
*A*_4_ (%)	21 ± 5	30 ± 16	*P* = 0.206
Max gain (%RSNA mmHg^−1^)	−2.94 ± 1.18	−2.53 ± 0.38	*P* > 0.999
Maximum response (%)	133 ± 9	137 ± 29	*P* > 0.999
MAP threshold (mmHg)	82 ± 14	93 ± 15	*P* = 0.123
MAP saturation (mmHg)	128 ± 31	136 ± 16	*P* = 0.645
Operating range (mmHg)	46 ± 26	42 ± 8	*P* > 0.999
**TNF‐α + CPZ (*n* = 6)**	**TNF‐α**	**TNF‐α+CPZ**	**TNF‐α vs. TNF‐α +CPZ**
*A* ^1^ (%)	87 ± 14	81 ± 14	*P* > 0.999
*A*_2_ (%RSNA mmHg^−1^)	0.10 ± 0.02	0.16 ± 0.05	*P* = 0.226
*A*_3_ (mmHg)	95 ± 18	100 ± 19	*P* = 0.336
*A*_4_ (%)	36 ± 6	38 ± 11	*P* > 0.999
Max gain (%RSNA mmHg^−1^)	−2.30 ± 0.75	−3.35 ± 1.29	*P* = 0.177
Maximum response (%)	123 ± 18	119 ± 15	*P* > 0.999
MAP threshold (mmHg)	76 ± 17	86 ± 17	*P* = 0.112
MAP saturation (mmHg)	115 ± 20	113 ± 22	*P* > 0.999
Operating range (mmHg)	40 ± 8.50	27 ± 8.39	*P* = 0.532
	**Vehicle vs. TNF‐α^a^**	**TNF‐α vs. TNF‐α+CPZ^a^**	
	*P* = **0.024**	*P* = 0.135	
	*P* = **0.035**	*P* = 0.059	
	*P* = 0.459	*P* = 0.304	
	*P* = 0.418	*P* = 0.656	
	*P* = 0.770	*P* = 0.486	
	*P* = 0.123	*P* = 0.305	
	*P* = 0.744	*P* = 0.709	
	*P* = 0.576	*P* = 0.223	
	*P* = 0.761	*P* = 0.263	

*Note*: Values are means ± SD. ^a^ Ddenotes comparisons made between groups during the second intrarenal infusion phase. *A*_1_, response range; *A*_2_, gain coefficient; *A*_3_, pressure at the midrange of the curve (centring point); *A*_4_, minimum response. Comparisons between vehicle, TNF‐α and TNF‐α + CPZ were carried out using repeated‐measure two‐way ANOVA followed by Tukey's and Bonferroni's *post hoc* tests where relevant. Significance is taken at *P* < 0.05. Abbreviations: CPZ, capsazepine; HR, heart rate; MAP, mean arterial pressure; RSNA, renal sympathetic nerve activity.

There were no significant changes in the HR baroreflex curve parameters during CPZ intrarenal infusion in the presence of a background intrarenal TNF‐α infusion (Supporting information, Figure S2: https://figshare.com/s/1f3cfbd9060f3b0d61ac).

## 4 DISCUSSION

The hypothesis tested in this study was that the mechanism of impaired arterial and cardiopulmonary baroreflex control in cisplatin‐induced renal injury in rats involves TRPV1 channels. Furthermore, inflammatory mediators in the kidney, particularly TNF‐α, play a key role in impaired baroreflex control through a mechanism involving TRPV1. The main finding of the present study was that the TRPV1 antagonist CPZ restored the arterial and cardiopulmonary baroreflex control of RSNA in cisplatin‐induced renal injury in the rat. The impaired baroreflex mechanism in this model might be linked to inflammatory mediators within the kidney, in particular TNF‐α, through a mechanism that partly involves TRPV1.

Cisplatin is a widely used chemotherapeutic agent, but its nephrotoxic effects are a major clinical concern, often leading to AKI and increased risk of chronic kidney disease (CKD) and mortality (Cornelison & Reed, 1993; McSweeney et al., 2021; Mizuno et al., 2012; Pabla & Dong, 2008). Both cardiovascular and autonomic dysregulation have been reported in human patients receiving cisplatin therapy, with possible involvement of the baroreflex (Boogerd et al., 1990; Hansen, 1990; Sagstuen et al., 2005; Viana‐Cardoso et al., 2011). The cisplatin rat model of renal injury on the other hand is associated with impaired baroreflex sensitivity, which has been linked to a derangement of the renal innervation (Goulding & Johns, 2015; Khan et al., 2014). The present study showed that the RSNA baroreflex was blunted 4 days after cisplatin injection. This was demonstrated by a lower curvature coefficient (*A*_2_) as well as maximum gain of the baroreflex function curve in the cisplatin group compared to the control. There was a significant increase in MAP saturation in the cisplatin group, associated with a greater operating range (MAP saturation–MAP threshold). Therefore, the blunted RSNA baroreflex in the cisplatin group was associated with a significant increase in MAP saturation and operating range and a decrease in the gain coefficient and maximal gain. Interestingly, the increase in MAP saturation in the cisplatin group was not correlated with the baseline MAP values, in contrast to the significant correlation seen in the control group (Figure 5). This suggests that the baroreflex changes are inherent to the cisplatin‐induced injury and present even at basal blood pressure levels.

The blunted blood pressure baroreflex regulation of RSNA during renal injury may be due to aberrant renal afferent nerve activation, leading to a sympatho‐excitation (Gauthier et al., 2023). Indeed, the renal afferent nerves play a critical role in modulating autonomic output through their projections to key regions of the central nervous system. Anatomical and functional studies have shown that renal afferents project to the nucleus tractus solitarius, the primary integration site for baroreflex signalling (Nishi et al., 2015; Solano‐Flores et al., 1997). Additionally, activation of renal afferents can modulate baroreflex sensitivity by influencing neuronal activity in the paraventricular nucleus (PVN), which in turn regulates sympathetic outflow (Pyner, 2009; Xu et al., 2015). This was further demonstrated by the increased expression of *Fos* gene, a marker of neuronal activation, in the PVN due to volume expansion in conscious rabbits (Akama et al., 1998). Ng et al. (2004) showed that inhibition of PVN neurones with the GABA agonist muscimol attenuated the sympathoinhibitory response to volume expansion. These findings highlight the importance of afferent‐driven modulation of central circuits in shaping cardiovascular and renal responses to volume changes.

The dysregulation in the afferent renal nerve mechanism seems to be driven by the TRPV1 channel found on these afferent nerves in the renal pelvis, cortex and medulla (AlMarabeh et al., 2019, 2021). A recent study by Stocker & Sullivan (2023) showed that deletion of TRPV1 channels in the 2K1C model of renal injury and hypertension in rats restored elevated renal afferent nerve activity and attenuated the degree of sympatho‐excitation and hypertension. Thus, TRPV1 plays a potential role in the blood pressure control mechanisms in renal injury and inflammation by mediating afferent renal nerve signalling. Accordingly, the hypothesis was tested that TRPV1 blockade would restore the arterial and cardiopulmonary baroreflex control of RSNA in cisplatin renal injury rats. The present study showed a blunted RSNA baroreflex control mechanism 4 days after cisplatin injection, which was restored following the blockade of renal TRPV1 using CPZ. The slope coefficient and maximum gain were both increased in the presence of CPZ. Additionally, the MAP saturation and operating range were both decreased to near control values in the presence of CPZ. Therefore, renal TRPV1 is involved in a mechanism that modifies the RSNA baroreflex in renal injury and inflammation.

CPZ in the present study was infused approximately at the corticomedullary border of the kidney. This approach allowed the diffusion of CPZ into the renal cortex, medulla and pelvic wall, where renal afferent nerves are located, as shown by the diffusion of lissamine green dye in previous studies from this laboratory (AlMarabeh et al., 2021; O'Neill et al., 2017). The dose of CPZ was also sufficient to block TRPV1 as a similar dose of CPZ blocked TRPV1 effects and resulted in a marked change in RSNA in a previous study (Li & Wang, 2006). Ditting et al. (2012) showed that stimulation of TRPV1 within the renal pelvis by capsaicin or by increasing renal pelvic pressure resulted in a dose‐dependent increase in afferent renal nerve activity which could be abolished by renal pelvic perfusion of CPZ. Additionally, a previous study in rats using the same dose of CPZ showed inhibition of TRPV1 effects in the kidney, as well as a blunted afferent renal nerve response to increasing intrapelvic pressure (Feng et al., 2008). More recently, we showed a blunted diuretic and natriuretic response to volume expansion in a rat model of chronic intermittent hypoxia (CIH). Intrarenal infusion of a similar dose of CPZ restored the diuretic and natriuretic responses to volume expansion in CIH, indicating a potential role of renal TRPV1 in mediating impaired renal excretory function in this model (AlMarabeh et al., 2021). Additionally, the restored diuretic and natriuretic responses during volume expansion in CPZ‐treated CIH rats were not accompanied by a significant change in the reflex sympatho‐inhibitory response. In contrast, in the present study, the sympatho‐inhibitory response to volume expansion was enhanced in response to intrarenal infusion of CPZ. However, it should be noted that the CIH model we utilised previously was associated with a mild increase in renal inflammatory markers compared to the cisplatin renal injury model. The cisplatin model in this study showed severe changes in renal injury and inflammation markers as reported in previous studies from this lab (Goulding & Johns, 2015) as well as by others (Li et al., 2023; Oh et al., 2014; Zhang et al., 2007). Together, our findings point to an important role for TRPV1 channels in the modulation of renal afferent nerve‐mediated baroreflex dysfunction in the cisplatin renal injury model that might be related to a higher level of local inflammatory mediators.

Higher levels of inflammatory mediators, in particular TNF‐α, were found in the kidney in cisplatin renal injury (Deng et al., 2001; Ramesh & Reeves, 2002, 2004; Zhang et al., 2007) as well as other renal injury and hypertension models including the deoxycorticosterone acetate (DOCA) hypertension model (Banek et al., 2016; Elmarakby et al., 2008), angiotensin II‐induced hypertension (Guzik et al., 2007) and salt‐sensitive hypertension (Huang et al., 2016) indicating a possible role of these mediators in initiating a sympatho‐excitatory response leading to impaired blood pressure homeostasis. The present study showed that the baroreflex sensitivity, presented as the gain coefficient, during intrarenal infusion of TNF‐α was significantly lower than in the presence of intrarenal saline infusion. The infusion of TNF‐α into the kidney of control rats in a previous study from this laboratory blunted the baroreflex control (Abdulla et al., 2019). Additionally, intrarenal pelvic infusion of TNF‐α enhanced the sympatho‐excitatory response to intrarenal pelvic administration of hypertonic NaCl and adenosine (Abdulla et al., 2024). Meanwhile, the inhibition of TNF‐α effects with tacrolimus restored the baroreflex control of RSNA in the cisplatin model of renal injury in a previous study from this group (Abdulla et al., 2019). Similarly, tacrolimus or total renal denervation restored the baroreflex control in a high‐fat diet‐induced obese rat model characterised by renal inflammation (Khan et al., 2017). Banek et al. (2016) found that the reduction in blood pressure of DOCA‐treated hypertensive rats following total renal denervation was associated with significantly lower levels of pro‐inflammatory markers within renal tissues, including TNF‐α, compared to sham‐treated animals. Together, these data provide evidence for the contribution of TNF‐α within the kidney in mediating renal nerve‐dependent derangement of the blood pressure control mechanisms in rats with renal injury.

The present study explored the mechanisms by which TRPV1 blockade using CPZ ameliorated the arterial and cardiopulmonary baroreflex mechanisms during cisplatin renal injury. TNF‐α is considered a key inflammatory mediator that is activated by cisplatin as blockade of TNF‐α was found to provide protection against cisplatin nephrotoxicity (Ramesh & Reeves, 2002). TNF‐α also enhances TRPV1 expression on sensory neurons of the dorsal root ganglia (Hensellek et al., 2007; Wang et al., 2018). To this end, TNF‐α in this study was infused at the corticomedullary border of the kidney before and during a background infusion of CPZ. The intrarenal infusion of TNF‐α in the present study was associated with an increase in both baseline RSNA and baroreflex curve mid‐point blood pressure (*A*_3_). The infusion of CPZ intrarenally with TNF‐α restored the increase in both integrated RSNA and *A*_3_. Furthermore, the gain coefficient of the RSNA baroreflex was slightly increased in the presence of CPZ. This points to a partial role for the TRPV1 in mediating the blunted arterial and cardiopulmonary baroreflex due to higher kidney tissue levels of TNF‐α. This notion is supported by our recent study in which intrarenal pelvic infusion of TNF‐α resulted in a slight increase in the sympatho‐excitatory response to TRPV1 activation by intrarenal pelvic infusion of capsaicin (Abdulla et al., 2024). Additionally, in another study from this laboratory we showed that bradyzide, a bradykinin type 2 receptor antagonist, restored the high‐pressure baroreflex control of RSNA in cisplatin‐induced renal injury rats (Abdulla et al., 2016). Bradykinin is a pro‐inflammatory mediator that has been extensively studied and shown to activate afferent renal nerves (Barry & Johns, 2015; Kopp et al., 1997; Smits & Brody, 1984). This sympatho‐excitatory response to bradykinin was found to be mediated in part by TRPV1 in the kidney in a preliminary study from this laboratory (Barry & Johns, 2012). Bradykinin can also induce the release of inflammatory cytokines, such as TNF‐α and IL‐1β, which are involved in cisplatin‐induced renal injury (Estrela et al., 2014; Tiffany & Burch, 1989). Results from this study and previous findings thus strengthen the hypothesis that inflammatory mediators are produced during renal injury and activate the afferent renal nerves, consequently resulting in an impaired baroreflex control of sympathetic nervous system. The present study examined the baroreflex mechanism utilising male rats. Indeed, previous studies in experimental animals (Caeiro et al., 2011; Johnson et al., 2011) and humans (Abdel‐Rahman et al., 1994; Tanaka et al., 2004) suggest sex‐based differences in baroreflex control. Notably, the increase in vagal outflow in response to baroreceptor activation is smaller in females compared to males, suggesting potential sex differences in baroreflex sensitivity (Abdel‐Rahman, 1999). Future studies should explore whether these sex‐based differences influence the observed effects of CPZ and TNF‐α.

### 4.1 Limitations

The study presents a number of limitations. First, the cisplatin model is a severe model of kidney injury and inflammation with direct effects on the immune system by promoting inflammatory responses and activation of immune cells (Galsky et al., 2024; Zhou et al., 2022). Additionally, cisplatin administration results in the upregulation of a number of cytokines including TNF‐α, IL‐1β, transforming growth factor β, monocyte chemoattractant protein 1, IL‐10, IL‐6 and IL‐11 (Mitazaki et al., 2009; Ramesh & Reeves, 2005; Zager et al., 2006). Therefore, multiple factors are involved in the mechanism of impaired baroreflex regulation in the cisplatin model of kidney disease. Our studies in normal rats utilising different doses of intrarenal TNF‐α pointed to a key role of this inflammatory mediator in enhancing the sympatho‐excitatory response of intrarenal pelvic infusion of hypertonic NaCl and adenosine (Abdulla et al., 2024). Similarly, TNF‐α inhibitors ameliorated cisplatin‐induced renal dysfunction and restored the blunted arterial and cardiopulmonary baroreflexes (Abdulla et al., 2019; Ramesh & Reeves, 2002). Likewise, TNF‐α‐deficient mice were resistant to cisplatin nephrotoxicity (Ramesh & Reeves, 2002). Therefore, although TNF‐α is the main regulator of the inflammatory response during cisplatin renal injury, further research is needed to investigate the roles of various other cytokines in this model. A second limitation is that intrarenal CPZ administration was restricted to a single kidney, which may not fully capture the effects of TRPV1 antagonism. Nevertheless, our study builds on recent findings in the CIH model of hypertension and renal injury where the blunted diuretic and natriuretic responses to volume expansion were restored using a similar approach (AlMarabeh et al., 2021). Further studies utilising bilateral TRPV1 inhibition are needed to explore these effects more comprehensively. Finally, the baroreflex mechanism in the present study was evaluated in anaesthetized rats, where the anaesthetic agents were found to alter its function. For example, urethane, which was used in this, is known to attenuate the tachycardic response to a decrease in blood pressure (Stornetta et al., 1987). Further investigations in conscious animals are needed to eliminate any potential effects of anaesthesia on the baroreflex responses in renal injury.

### 4.2 Conclusions

Taken together, the main finding of our study is that the TRPV1 antagonist CPZ restored the arterial and cardiopulmonary baroreflex control mechanisms that are impaired in cisplatin‐induced renal injury rats. These findings point to a possible deranged TRPV1‐mediated afferent renal nerve mechanism in the cisplatin renal injury model. The mechanism by which the baroreflexes are impaired in this model involves TNF‐α, with partial involvement from TRPV1. The present study may provide further insight into the mechanisms underlying impaired baroreflex control during renal injury.

## SUPPORTING INFORMATION

Supporting information can be found at Figshare.

https://figshare.com/s/51ea85fc5ca7220ce1a1

Table S1. Baseline values in the volume expansion experiment

Table S2. Heart rate baroreflex parameters in the cisplatin study

https://figshare.com/s/1f3cfbd9060f3b0d61ac

Figure S1. Heart rate baroreflex gain curves in the cisplatin study

Figure S2. Heart rate baroreflex gain curves in the TNF‐α study

## AUTHOR CONTRIBUTIONS

Mohammed H. Abdulla and Edward J. Johns conceived the idea and designed the study; Mohammed H. Abdulla performed in vivo experiments with the assistance of Ella Murphy and Lauren Mulcahy; Mohammed H. Abdulla, Ella Murphy and Lauren Mulcahy analysed and interpreted data; Mohammed H. Abdulla drafted the manuscript with revisions from Edward J. Johns All authors have read and approved the final version of this manuscript and agree to be accountable for all aspects of the work in ensuring that questions related to the accuracy or integrity of any part of the work are appropriately investigated and resolved. All persons designated as authors qualify for authorship and all those who qualify for authorship are listed.

## CONFLICT OF INTEREST

None declared.

## FUNDING INFORMATION

None.

## Data Availability

The data that support the findings of this study are available on request from the corresponding author.
